# Research on a hybrid neural network task assignment algorithm for solving multi-constraint heterogeneous autonomous underwater robot swarms

**DOI:** 10.3389/fnbot.2022.1055056

**Published:** 2023-01-10

**Authors:** Jingyu Ru, Dongqiang Hao, Xiangyue Zhang, Hongli Xu, Zixi Jia

**Affiliations:** Faculty of Robot Science and Engineering, Northeastern University, Shenyang, China

**Keywords:** task assignment problem, multiple autonomous underwater robots, cluster collaboration, genetic algorithm, graph pointer network

## Abstract

Studying the task assignment problem of multiple underwater robots has a broad effect on the field of underwater exploration and can be helpful in military, fishery, and energy. However, to the best of our knowledge, few studies have focused on multi-constrained underwater detection task assignment for heterogeneous autonomous underwater vehicle (AUV) clusters with autonomous decision-making capabilities, and the current popular heuristic methods have difficulty obtaining optimal cluster unit task assignment results. In this paper, a fast graph pointer network (FGPN) method, which is a hybrid of graph pointer network (GPN) and genetic algorithm, is proposed to solve the task assignment problem of detection/communication AUV clusters, and to improve the assignment efficiency on the basis of ensuring the accuracy of task assignment. A two-stage detection algorithm is used. First, the task nodes are clustered and pre-grouped according to the communication distance. Then, according to the clustering results, a neural network model based on graph pointer network is used to solve the local task assignment results. A large-scale cluster cooperative task assignment problem and a detection/communication cooperative work mode are proposed, which transform the cooperative cooperation problem of heterogeneous AUV clusters into a Multiple Traveling salesman problem (MTSP) for solving. We also conducted a large number of experiments to verify the effectiveness of the algorithm. The experimental results show that the solution efficiency of the method proposed in this paper is better than the traditional heuristic method on the scale of 300/500/750/1,000/1,500/2,000 task nodes, and the solution quality is similar to the result of the heuristic method. We hope that our ideas and methods for solving the large-scale cooperative task assignment problem can be used as a reference for large-scale task assignment problems and other related problems in other fields.

## 1. Introduction

With the development of underwater vehicle technology and information technology, new underwater detection needs are constantly emerging. Under the constraints of multi-agents, more challenges are emerging, and different scholars have focused on related research directions. The assignment of detection tasks is a relatively classic research direction when using AUV clusters to perform traversal detection of multiple points to be detected in underwater detection scenarios.

The task assignment of underwater detection robots can be divided into two types: dynamic task assignment and static task assignment, which correspond to different usage scenarios. When performing detection tasks on dynamic targets (Page et al., 2010; Xie et al., 2018), the task allocation method of dynamic allocation is often used because the situation of the area to be detected is unknown at this time, detection tasks always appear, and tasks can only be allocated while exploring. Many scholars have focused in this topic. For example, MahmoudZadeh et al. (2018) proposed a hierarchical dynamic task planning framework for the problem of dynamic task assignment of AUVs within a limited time interval in a spatiotemporally changing marine environment. Bertuccelli et al. (2009) proposed a dynamic mission planning algorithm based on enhanced Consensus-Based Bundle Algorithm for multi-agent combat scenarios with noisy sensors. Capitan et al. (2016) proposed a dynamic task planning algorithm based on MDP (Markov Decision Process) for planning problems under multi-stage uncertainty. The above problems have no global information, and the task allocation will focus on factors, such as the robot's detection ability, communication delay, and energy allocation. When assigning static tasks (Ferreira et al., 2007; Edison and Shima, 2011), the related problem is usually modeled as a traveling salesman problem. For example, Abbasi et al. (2022) proposed a heuristic fleet cooperation algorithm to solve the problem of sea star cluster processing. Hooshangi and Alesheikh (2017) explored a multi-agent task planning method combining interval number VIKOR and auction mechanism based on Contract Net Protocol is used to solve rescue problems in disaster environments. In addition, many scholars have used deep learning (Vinyals et al., 2015; Bello et al., 2016; François-Lavet et al., 2016; Deudon et al., 2018; Kool et al., 2018; Holler et al., 2019; Solozabal et al., 2019) methods to solve the traveling salesman problem. The above work uses more capable surface and underwater ships to pre-scan the area to be detected, a more capable experimental platform to improve some of the above shortcomings in detection and energy consumption, and a multi-robot cluster to perform the detection task, but requires a large number of AUVs that can perform communication and detection tasks. The cost is high and the number is small. The number of task points allocated to each AUV is large, and the computational efficiency and detection efficiency of the task allocation algorithm are relatively low. Thus far, the underwater task detection task still faces many problems, and limited research has focused on task assignments for large-scale detection points in the pre-detection area using the heterogeneous AUV cluster combination of communication/detection.

Studying the task assignment problem of heterogeneous AUV cluster combinations for large-scale probe points can bring many benefits (Zhu et al., 2020; Ru et al., 2021). In terms of energy consumption, the heterogeneous AUV combination performs its duties, which can provide smaller energy consumption and prolong the working time of the AUV cluster (Zhu et al., 2017; Khan et al., 2022). In terms of task allocation efficiency, the AUV responsible for communication has strong computing power and can be equipped with deep learning modules. It can greatly improve the efficiency of task allocation (Zhu et al., 2019; Khan and Li, 2022). In terms of economy, the types and performance of sensors configured by small robots that perform short-range detection tasks are weak, and the cost is low. It can be used in combination with large AUVs with strong detection capabilities to save costs (Huang et al., 2014; Khan et al., 2021). In terms of detection efficiency, heterogeneous clusters can detect more detection points per unit time and increase the detection area per unit time (Li et al., 2017).

Heterogeneous AUV cluster detection with detection/communication hybrid functions has many benefits but still faces the following challenges. First, the balance of robot task allocation is an issue considering that the number of points obtained by pre-detection increases with the increase of sensor capabilities and detection requirements. How to reasonably allocate detection points to each robot group is another challenge. The second is the cooperation between heterogeneous robots. Because the functions of heterogeneous robots are different, robots with functions such as detection and communication need to cooperate in the time and space domains, so the cooperation between heterogeneous robots is a challenge. Finally, the multi-robot task assignment problem is a typical NP-hard problem, and the efficient assignment of tasks is a challenge.

To overcome the above challenges, we propose a novel task assignment method suitable for solving heterogeneous AUV cluster combinations-Cluster-based hybrid solution method: This algorithm (i) proposes a detection point assignment method, (ii) designs a set of task assignment algorithm based on the fusion of GPN (Ma et al., 2019) network and heuristic method, and (iii) proposes a heterogeneous AUV matching algorithm. The contributions of this paper are as follows:

(1) To our knowledge, this paper is the first to use the area detection algorithm in a large-scale underwater environment to be detected by using a heterogeneous segmentation method.

(2) A DBSCAN clustering equivalence algorithm based on communication distance constraints that can perform grouping equivalent processing on large-scale tasks is proposed.

(3) An improved task assignment method based on GPN network is also proposed, which can effectively replace the traditional heuristic algorithm to solve the TSP problem with fixed start and end points.

(4) A task coordination method for heterogeneous AUVs that can work under the common constraints of detection and communication for heterogeneous AUV systems is explored.

(5) We also carried out a large number of simulation experiments on virtual underwater pre-detection points, compared the effects of classical heuristic algorithms, and analyzed the combination of different numbers of robots to further verify the effectiveness and efficiency of the algorithm practicality.

## 2. Problem description

There are *N* target points *tp*_*i*_ to be processed in a certain sea area, forming a set of tasks to be processed *TP* :


(1) $$\begin{array}{l} TP=\left\{tp_{1},tp_{2},\ldots ,tp_{i},\ldots ,tp_{N}\right\}\;1\leq i\leq N \end{array}$$

where *tp*_*i*_ = {*x, y*}, *x, y* represents the location information of the target task point.

Existing *M*_1_ communication units *cu*, and *M*_2_ execution units *eu* constitute cluster unit *U* :


(2) $$\begin{array}{l} U=\left\{cu_{1},cu_{2},\ldots ,cu_{i},\ldots ,cu_{M1},eu_{1},eu_{2},\ldots ,eu_{j},\ldots ,eu_{M2}\right\} \end{array}$$

When the execution unit and the communication unit cooperate to access all task nodes, they should meet the communication constraint requirements, as shown in Equation (3), that is, the execution unit should be within the scope of the communication unit. In addition, the execution unit should also meet the requirements of its own capability constraints, as shown in Equation (4), that is, at the same time, the execution unit can only access at most one target task node. The specific constraints are as follows:


(3) $$C_{i,j}=\{\begin{array}{cc} 0, & d_{i,j}\leq r\;\\ 1, & d_{i,j}>r \end{array}$$

where *C*_*i,j*_ indicates whether communication can be established between communication unit *cu*_*i*_ and execution unit *eu*_*j*_, *C*_*i,j*_ = 1 indicates that the communication unit can establish communication with the execution unit, and vice versa, *d*_*i,j*_ represents the distance between the communication unit *cu*_*i*_ and the execution unit *eu*_*j*_, and *r* represents the communication radius of the communication unit *cu*_*i*_.


(4) $$\begin{array}{l} \overset{N}{\underset{i=1}{\sum }}h\left(eu_{j},tp_{i},t\right)\leq 1 \end{array}$$

where *h*(*eu*_*j*_, *tp*_*i*_, *t*) indicates whether the execution unit *eu*_*j*_ accesses the target task point *tp*_*i*_ at time *t*, the value of *h*(*eu*_*j*_, *tp*_*i*_, *t*) is 1 if the execution unit *eu*_*j*_ visits the target task point *tp*_*i*_, and 0 otherwise.

In order to ensure the optimal result of the overall task assignment, this study takes the minimum moving distance as the optimization goal to optimize the entire task assignment process. The optimization goals are as follows:


(5) $$\begin{array}{l} f=\overset{M_{1}}{\underset{i=1}{\sum }}L_{cu_{i}}+\overset{M_{2}}{\underset{j=1}{\sum }}L_{eu_{j}} \end{array}$$

where *L*_*c*_*u*__*i*__ represents the total distance moved by the communication unit *cu*_*i*_, and *L*_*e*_*u*__*j*__ represents the total distance moved by the execution unit *eu*_*j*_.

## 3. Cluster collaborative task assignment solution framework

**Figure 1:**
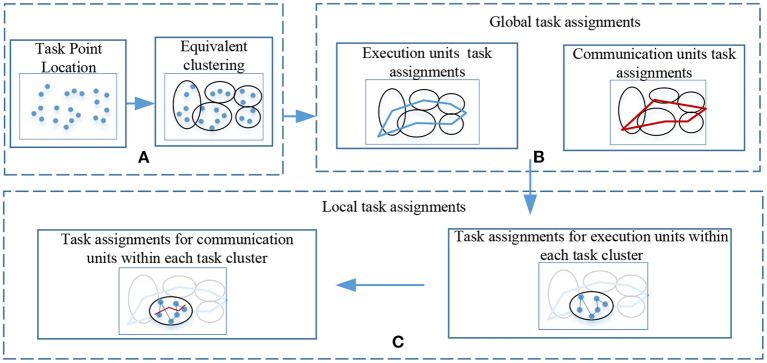
Flowchart for solving cluster collaborative task assignment. **(A)** Perform equivalent clustering on all task nodes, and generate several task cluster units after clustering. **(B)** Perform global task assignment of execution units and communication units according to the equivalent clustering results. **(C)** According to The result of the global task assignment is to assign tasks to the communication unit and the execution unit within the task cluster.

The execution and the communication units need to cooperate to complete the processing of all task points, and a communication distance constraint between the execution and the communication units exists, limited by the current computing power level. Hence, it is difficult to directly solve the task assignment and solve it in a limited time. For optimal task assignment results, the process of solving the cluster cooperative task assignment problem in this paper is shown in the following Figure 1.

Module A means to perform equivalent clustering on all task points, module B means to plan the order in which the execution unit and communication unit access each task cluster according to the clustering result, module C means to allocate within each task cluster according to the global task allocation result local tasks.

### 3.1. Target task point clustering grouping

Considering the influence of communication constraints, the execution unit must select executable task points near the communication unit. At the same time, when the task scale becomes larger, the overall optimization will become more complicated. Therefore, consider grouping tasks first through communication distance constraints, and then, large-scale tasks and resource allocation planning problems become local small-scale problems, thereby reducing the amount of computation. The grouping method adopts the DBSCAN method to group the task points:


(6) $$\begin{array}{l} TP=\left\{g_{1},g_{2},\ldots ,g_{i},\ldots ,g_{k}\right\}\;1\leq k\leq N \end{array}$$

where *g*_*i*_ = {*tp*_1_, *tp*_2_, …, *tp*_*l*_} indicates that the task cluster *g*_*i*_ has *l* target task points, and *k* represents the number of task clusters after clustering.

First, according to the distribution of target task points *tp*_*i*_ in the task cluster group *g*_*i*_, it is equivalently converted into a node, and then the equivalent approximation is made to the moving distance and time consuming of the execution unit *eu*_*j*_ to complete the task cluster.

Task cluster *g*_*i*_ equivalent node *E*_*x,y*_(*g*_*i*_) location coordinates is as follow:


(7) $$\begin{array}{l} E_{x,y}\left(g_{i}\right)=\min f_{r}\left(g_{i}\right) \end{array}$$

where *f*_*r*_(*g*_*i*_) represents the center coordinates of the smallest covering circle containing all target task points *tp*_*i*_ in the task cluster *g*_*i*_.

The equivalent approximate moving distance *E*_*d*_ of the execution unit after visiting all target task point *tp*_*i*_ in the task cluster *g*_*i*_ is as follow:


(8) $$\begin{array}{l} E_{d}=\overset{l}{\underset{i=1}{\sum }}∥E_{x,y}-tp_{i}{∥}_{2} \end{array}$$

The equivalent approximate time *E*_*t*_ for the execution unit to complete the task cluster *g*_*i*_ is as follows:


(9) $$\begin{array}{l} E_{t}=\frac{E_{d}}{\bar{v}} \end{array}$$

where $\bar{v}$ represents the average expected speed of execution unit.

### 3.2. Global task assignment

#### 3.2.1. Execution unit task assignment

According to the clustering and grouping results *g*_*i*_, the global task assignment problem of execution units can be transformed into a multi-travel salesman problem with fixed start and end nodes. Genetic algorithm is used to solve the optimal task cluster access sequence of each execution unit *eu*_*j*_, and the minimum moving data distance is used as the The optimization objective of the problem optimizes the task assignment results. The specific form of the optimization goal is as follows:


(10) $$\begin{array}{l} f=\overset{M_{2}}{\underset{j=1}{\sum }}L_{eu_{j}} \end{array}$$

#### 3.2.2. Communication unit task assignment

The communication unit needs to cooperate with the execution unit to complete the processing of task points and assign tasks to the communication unit according to the global planning result of the execution unit. The time required by the execution unit to process each task cluster also varies because of the different number of tasks in each task cluster. Therefore, the following time constraints exist for the communication unit to reach each task cluster node:


(11) $$\begin{array}{l} a_{j}+w_{j}+{\text{Δ}}_{i,j}\leq e_{i,j} \end{array}$$

where *w*_*j*_ = max(0, *e*_*i*_−*a*_*j*_), *a*_*j*_ is the time when the communication unit arrives at the task cluster node, *w*_*j*_ is the waiting time of the communication unit, *e*_*i,j*_ is the time when the ith execution unit starts to execute the jth node, and Δ_*i,j*_ is the time when the communication unit arrives from node i to node j.

The global task assignment problem of communication units can be equivalently transformed into a multi-travel salesman problem with time windows. In order to ensure that the optimal task assignment results of communication units are obtained, this paper takes the minimum moving distance of communication units as the optimization objective, and adopts genetic The algorithm solves the problem. The optimization goal is defined as:


(12) $$\begin{array}{l} f=\overset{M_{2}}{\underset{i=1}{\sum }}L_{eu_{i}} \end{array}$$

### 3.3. Local task assignment

During the execution of the task, the communication unit does not participate directly in the processing of the task point and is only responsible for completing the communication with the execution unit, that is, it does not need to reach the task point. In group task planning, a genetic algorithm is used to plan tasks for execution units, and then tasks are planned for communication units according to the results of task planning for execution units.

#### 3.3.1. Execution unit local task planning

**Figure 2:**
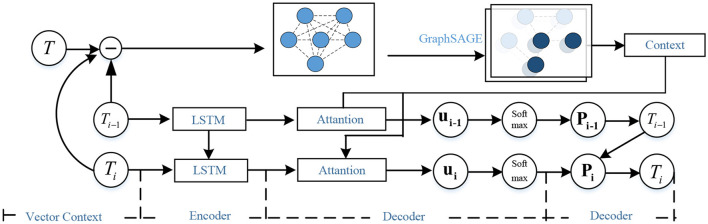
GPN network structure model diagram.

The local task assignment problem of the execution unit belongs to the traveling salesman problem with fixed start and end points. In this paper, the deep learning method based on the GPN model (Ma et al., 2019) is used to solve the local task assignment problem. The model structure is shown in the Figure 2.

The encoding part of the model is divided into node feature information encoding and neighbor node information encoding. The node location feature information is encoded through the LSTM network, thereby mapping the node feature information from the low-dimensional space to the high-dimensional space. According to the encoding vector of the location features of each node by LSTM, the node neighbor information encoding part aggregates and encodes the neighbor information of each node through the GraphSAGE network, so as to obtain the feature information between the node and other nodes. The network form of each layer of the neighbor node information encoding network is as follows:


(13) $$\begin{array}{l} {\text{T}}_{i}^{l}=\gamma {\text{T}}_{i}^{l-1}\text{Θ}+\left(1-\gamma \right)R_{\theta }\left(\frac{1}{\left|N\left(i\right)\right|}{\left\{{\text{T}}_{j}^{l-1}\right\}}_{j\in N\left(i\right)\cup \left\{i\right\}}\right) \end{array}$$

In the formula, ${\text{T}}_{i}^{l}$ represents the *l*−*th* task node of the layer, γ is a trainable weight matrix, $\text{Θ}\in {\mathbb{R}}^{d_{l-1}*d_{l}}$ is a trainable weight matrix, *R*_θ_ represents the aggregation function, and *N*(*i*) represents the *k* adjacent task nodes **T**_*i*_.

The decoder part encodes the node feature information and the neighbor node feature information to obtain the high-dimensional feature vector of the node and the high-dimensional feature vector of the neighbor node and send it to the attention network model to obtain the pointer vector *u*_*i*_, which is then passed to the softmax layer, using to generate the probability distribution *P*_*i*_ of the next node to visit.

#### 3.3.2. Local task planning of communication unit

**Figure 3:**
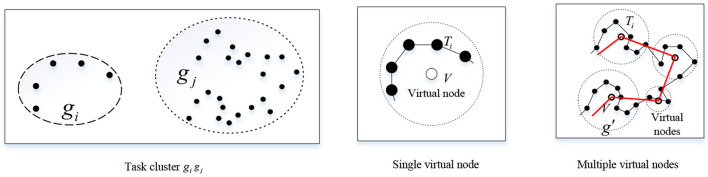
Schematic diagram of virtual node types.

Because the communication unit does not need to reach the task location point, virtual nodes *v*_*x, y*_ are added according to the task location point processed by the execution unit to plan the access node location of the communication unit. The types of virtual node additions are as follows Figure 3.

Single virtual nodemodel. When all target task points *tp*_*i*_ in the task cluster *g*_*i*_ are within the communication range of the communication unit *cu*_*i*_ that executes the task cluster, the virtual node *v*_*x, y*_ is defined as:


(14) $$\begin{array}{l} v_{x,y}=\min f_{r}\left(g_{i}\right) \end{array}$$

The above formula indicates that the coordinates of the virtual node *v*_*x,y*_ at this time are the center coordinates of the smallest covering circle containing all target task points in the task cluster *g*_*i*_.

Multiple virtual nodes model. When some target task points *tp*_*j*_ in the task cluster *g*_*i*_ are all within the communication range of the communication unit *cu*_*i*_ executing the task cluster, the task points of the current task group are grouped twice according to the order of the execution unit *eu*_*j*_ executing the task nodes.


(15) $$\begin{array}{l} g_{i}=\left\{g_{1}^{\prime },g_{2}^{\prime },\ldots ,g_{i}^{\prime },\ldots ,g_{a}^{\prime }\right\} \end{array}$$

where $g_{i}^{\prime }=\left\{tp_{j}|d_{i,j}\leq r\right\}$, $g_{i}^{\prime }$ represents a new small task cluster formed by re-clustering the task points *tp*_*i*_ in the task cluster unit *g*_*i*_ according to the communication range of the communication unit *cu*_*i*_, *a* represents the number of new task clusters generated by secondary clustering of the task cluster. At this point the virtual node looks like this:


(16) $$\begin{array}{l} v_{x,y}=\min f_{r}\left(g_{i}\right) \end{array}$$

To sum up, for the task assignment problem of underwater autonomous vehicles in multi-heterogeneous clusters, firstly, clustering is performed according to the location information of all task nodes, and the clustering results are equivalently approximated, and then the global tasks of the execution units are assigned. The problem is transformed into a multi-travel salesman problem to solve, and the communication unit task cooperative assignment problem is transformed into a multi-travel salesman problem with a time window to solve. Task allocation, the specific method is: the task allocation problem of the execution unit is transformed into the traveling salesman problem, which is solved by the deep learning method based on GPN, and the communication unit performs local task allocation by adding virtual nodes. The notation used in the design is summarized in Table 5.

## 4. Experiment

This paper uses an NVIDIA RTX2080 GPU to train the FGPN model, limited by memory size constraints. The training batch size is B = 50, the tsp scale size is size = 60, and 1,000 rounds of training are performed. The training time for each round is about 3 min. The rest of the algorithms are implemented based on MATLAB2019, and the device CPU model is Intel (R) Core (TM) i7-6500U@2.50GHz.

Experiment 1: Comparison of task allocation algorithms for individual execution unit *eu* in target task nodes *tp* of different scales in the 1*1 km area. The results are shown in Table 1.

**Table 1:** Compare the task assignments of target nodes with fixed start and end points of different sizes in the 1*1 km area.

**Method**	**TP10**		**TP20**	
	**Total Len**.	**Time**	**Total Len**.	**Time**
GA (Approximate Time)	2.4997	0.0184	4.0933	0.0497
GA (Approximate Len.)	2.7533	0.0195	4.1401	0.0485
FGPN	2.7515	0.0181	4.1663	0.0471
**Method**	**TP30**		**TP40**	
	**Total Len**.	**Time**	**Total Len**.	**Time**
GA (Approximate Time)	6.1451	0.0534	7.9638	0.0729
GA (Approximate Len.)	4.8887	0.1553	5.2182	0.6204
FGPN	4.995	0.0563	5.4861	0.0724
**Method**	**TP50**		**TP60**	
	**Total Len**.	**Time**	**Total Len**.	**Time**
GA (Approximate Time)	10.0089	0.0919	12.4997	0.1007
GA (Approximate Len.)	5.8712	0.8093	6.4742	1.1452
FGPN	5.9229	0.0926	6.3551	0.1099

The total Len. in the table is 10^3^*m* and the time unit is seconds. Total Len., total length; TP, target task points.

**Figure 4:**
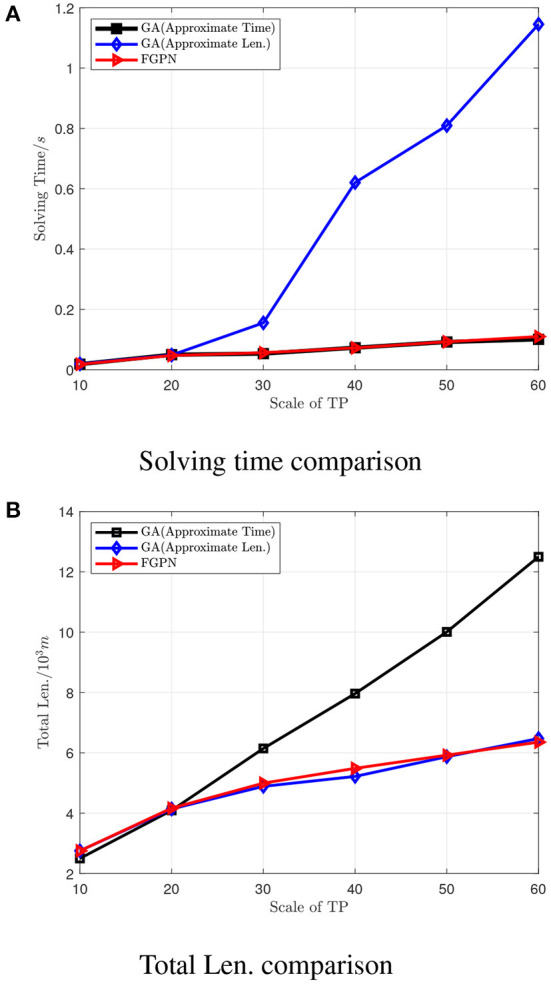
Comparison of solution times for the number of target task nodes at different scales. **(A)** Comparison of solution speed between GA algorithm and FGPN method when the solution results are similar. **(B)** Comparison of the solution results of the GA algorithm and the FGPN method when the solution speed is similar.

It can be seen from Figure 4 that the number of *TP* is less than 20, the solution results based on the deep learning method are similar in quality to the results obtained by GA, and the solution time is roughly the same; when the number of task nodes is greater than 20, the solution time is roughly the same. The quality of the solution based on the deep learning method is better than that of the GA solution. When the number of task nodes is greater than 40, the quality of the solution is improved by more than 30%. Moreover, when the number of task nodes is greater than 20, the quality of the solution is roughly under the same conditions, and the solution efficiency based on deep learning is better than that of GA. When the scale of task nodes is greater than 40, the solution efficiency is improved by about 70%.

Experiment 2: Comparison of results of task assignment methods based on the DBSCAN clustering method. Let the area size be 10*10km, the number of execution unit *eu* is 3, the number of communication unit *cu* is 2, the communication distance is 300 m, the movement speed of the execution unit is 2*m*/*s*, and the movement speed of the communication unit is 3 to 5 m/s. The results are shown in Tables 2–4.

**Table 2:** The relationship between the solution time and the total moving distance and the scale of the task nodes when the number of target task nodes in the task cluster is about 40. The unit of total Len. in the table is 10^6^*m*, and the unit of Time is seconds.

**Method**	**TP300**		**TP500**		**TP750**	
	**Total Len**.	**Time**	**Total Len**.	**Time**	**Total Len**.	**Time**
GA	0.24017	22.1086	0.6079	30.8226	1.1183	40.7717
BAS	0.7723	3.7901	1.9852	5.9216	3.5989	7.8401
FGPN	0.3141	3.3769	0.6284	5.277	1.4038	6.9828
**Method**	**TP1000**		**TP1500**		**TP2000**	
	**Total Len**.	**Time**	**Total Len**.	**Time**	**Total Len**.	**Time**
GA	1.4807	54.1713	2.7204	67.4411	3.9782	97.8231
BAS	4.7572	13.3097	8.7563	24.5971	13.7864	59.7851
FGPN	1.7045	11.8467	3.0505	21.8675	4.3854	55.2984

**Table 3:** The relationship between the solution time and the total moving distance and the scale of the task nodes when the number of target task nodes in the task cluster is about 50. The unit of total Len. in the table is 10^6^*m*, and the unit of Time is seconds.

**Method**	**TP300**		**TP500**		**TP750**	
	**Total Len**.	**Time**	**Total Len**.	**Time**	**Total Len**.	**Time**
GA	0.1864	21.672	0.5319	25.2549	0.8661	35.4905
BAS	0.8187	4.0607	1.7659	5.8291	3.8779	7.4967
FGPN	0.3115	3.6262	0.6073	5.2044	1.0134	6.6926
**Method**	**TTP1000**		**TP1500**		**TP2000**	
	**Total Len**.	**Time**	**Total Len**.	**Time**	**Total Len**.	**Time**
GA	1.6602	53.0959	2.2274	72.9084	3.8515	97.3034
BAS	5.5238	12.1733	8.392	23.6284	12.7955	56.2864
FGPN	1.7178	10.8701	2.2387	21.0986	4.0565	45.7959

**Table 4:** The relationship between the solution time and the total moving distance and the scale of the task nodes when the number of target task nodes in the task cluster is about 60. The unit of total Len. in the table is 10^6^*m*, and the unit of Time is seconds.

**Method**	**TP300**		**TP500**		**TP750**	
	**Total Len**.	**Time**	**Total Len**.	**Time**	**Total Len**.	**Time**
GA	0.24914	15.3671	0.44992	20.6146	0.79347	26.5818
BAS	0.8124	3.2167	1.5062	5.1463	3.8514	7.0196
FGPN	0.3348	3.1385	0.5722	4.9957	1.1201	6.8041
**Method**	**TP1000**		**TP1500**		**TP2000**	
	**Total Len**.	**Time**	**Total Len**.	**Time**	**Total Len**.	**Time**
GA	1.4785	53.2567	2.0076	70.3005	3.0399	89.8016
BAS	4.9487	10.6239	8.2158	20.5983	12.1072	54.3951
FGPN	1.4682	10.3123	2.3365	19.9997	3.7372	42.1359

**Table 5:** Variable explanation table.

**Variable name**	**Variable description**	**Variable name**	**Variable description**
*tp*_*i*_	i-th task point	*x, y*	The location information of task point
*cu*	Communication units	*eu*	Execution units
γ	Trainable weight matrix	*d*_*i,j*_	The distance between the *cu*_*i*_ and the *eu*_*j*_
*w*_*j*_	The waiting time of the *cu*_*j*_	*a*_*j*_	Time of arrival of *cu*
Θ	Trainable weight matrix	*e*_*i,j*_	The time when *eu*_*i*_ starts executing task
*R*_θ_	The aggregation function	Δ_*i,j*_	Time spent by *cu* on the way
*N*(*i*)	The *k* adjacent task nodes	${\text{T}}_{i}^{l}$	The *l*−*th* task node of the layer
*v*_*x,y*_	Virtual node		

Taking 1,000 task nodes in a 10*10 km area as an example, the overall task planning results are shown in the following figures.

**Figure 5:**
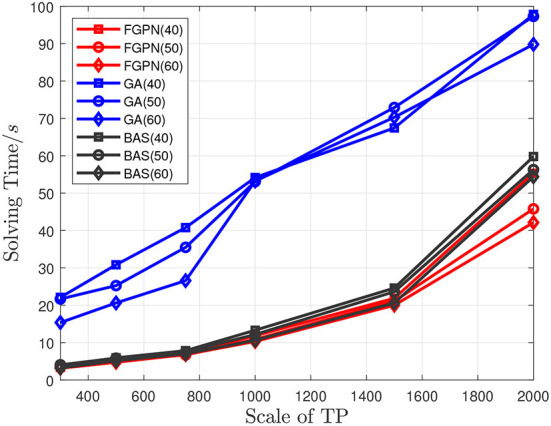
Comparison between the solution time of the three methods and the scale of task nodes when the number of target task nodes in the task cluster is 40/50/60.

**Figure 6:**
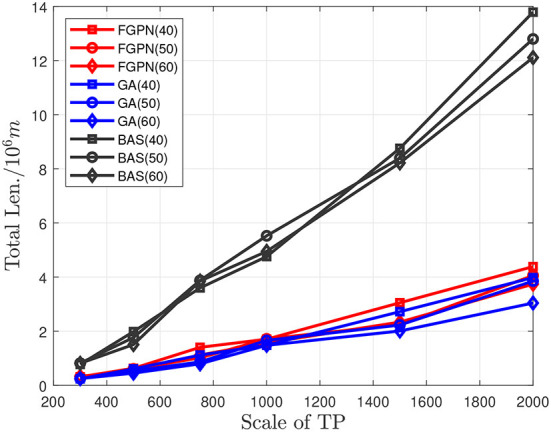
Comparison between the total length of the three methods and the scale of task nodes when the number of target task nodes in the task cluster is 40/50/60.

The experimental results indicate that in the 10*10 km area when the number of *TP* is between 300 and 500, as shown in Figures 5, 6, the solution time based on the deep learning method is similar to the total moving distance based on the solution result of the genetic algorithm, and the solution speed is increased by about 50%. When the task scale is greater than 500, the solution efficiency based on the deep learning method is better than that based on the genetic algorithm. When the total moving distance obtained by the solution remains similar, the solution speed is increased by more than 70%. Meanwhile, when the number of task nodes in the task cluster increases, the time spent to solve the relative optimal solution of the current scale task is relatively reduced, and when the scale of task nodes is greater than 1,500, it increases by about 20%. In addition, the solution efficiency of the BAS (Beetle Antennae Search Algorithm) is roughly similar to that of our proposed method, but the solution result is far worse than the genetic-based method and the method proposed in this paper. Experiments show that the method proposed in this paper can greatly improve the efficiency of solving large-scale cluster coordination problems.

**Figure 7:**
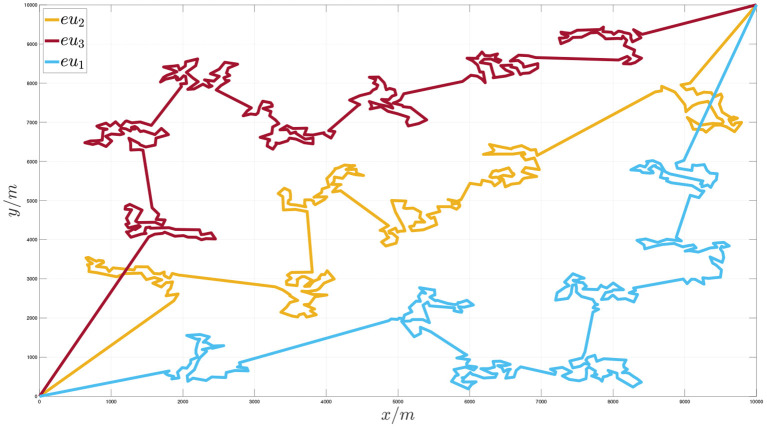
Schematic diagram of the execution unit accessing node sequence when the number of target task nodes is 1,000.

**Figure 8:**
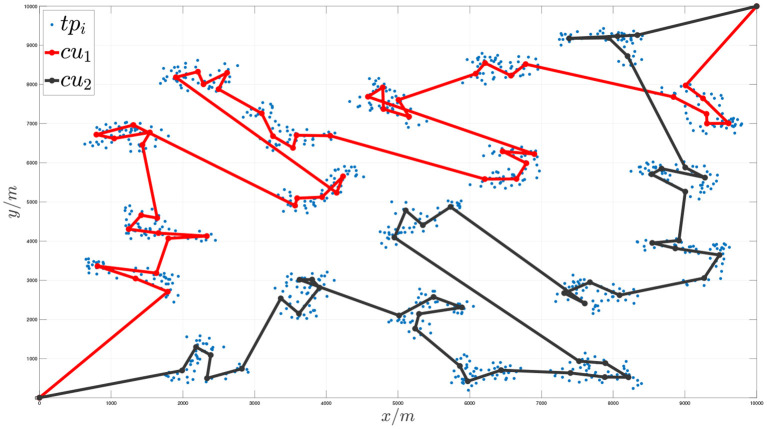
Schematic diagram of the communication unit cooperative access node sequence when the number of target task nodes is 1,000.

**Figure 9:**
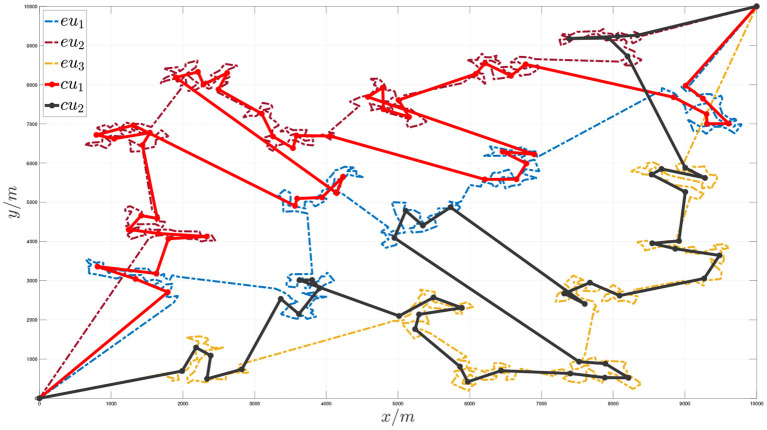
Schematic diagram of the communication unit and the execution unit cooperating to access the target task points.

Figures 7, 8, respectively, show the situation of three execution units traversing 1,000 task nodes and two communication units traversing virtual nodes cooperatively. Figure 9 shows the sequence of cooperative access to all target task nodes by the execution unit and the communication unit. Each execution unit traverses all the task nodes in the graph in turn, and the communication unit synchronously plans to traverse the virtual nodes of the graph according to the order in which the execution units access the task nodes to jointly complete the entire task. It can be seen from the figure that the algorithm proposed in this paper can effectively solve the problem of communication constraints and cooperative task assignment of multiple heterogeneous clusters.

## 5. Conclusion

This paper proposes a deep learning method and a heuristic algorithm by adopting the idea of divide and conquer and the combination of global and local, aiming at the large task scale and complex coordination difficulties in the large-scale cooperative task assignment problem of multi-heterogeneous cluster units with communication distance constraints. The FGPN method proposed in this paper, which combines the clustering-based GPN and the genetic algorithm, can greatly improve the solution efficiency while ensuring that the solution results are similar to the genetic algorithm when the number of target task nodes is between 1,000 and 1,500. The experimental results show that the algorithm proposed in this paper can solve the problem of cooperative assignment of large-scale cluster tasks and can obtain relatively optimal task assignment results faster while ensuring that the quality of the solution is roughly the same as that of the traditional method. We will further explore the use of deep learning methods to solve the multi-traveling salesman problem with fixed start and end positions and the multi-traveling salesman problem with time windows in the future.

## Data availability statement

The raw data supporting the conclusions of this article will be made available by the authors, without undue reservation.

## Author contributions

JR contributed to the conception of the study and contributed significantly to analysis and manuscript preparation. DH performed the experiment and performed the data analyses and wrote the manuscript. XZ, HX, and ZJ helped perform the analysis with constructive discussions. All authors contributed to the article and approved the submitted version.

## References

[B1] Abbasi A. MahmoudZadeh S. Yazdani A. (2022). A cooperative dynamic task assignment framework for COTSbot AUVs. IEEE Trans. Automat. Sci. Eng. 19, 1163–1179. 10.1109/TASE.2020.3044155

[B2] Bello I. Pham H. Le Q. V. Norouzi M. Bengio S. (2016). Neural combinatorial optimization with reinforcement learning. arXiv preprint arXiv:1611.09940. 10.48550/arXiv.1611.09940

[B3] Bertuccelli L. Choi H.-L. Cho P. How J. (2009). “Real-time multi-UAV task assignment in dynamic and uncertain environments,” in AIAA Guidance, Navigation, and Control Conference (Chicago, IL), 5776. 10.2514/6.2009-5776

[B4] Capitan J. Merino L. Ollero A. (2016). Cooperative decision-making under uncertainties for multi-target surveillance with multiples UAVs. J. Intell. Robot. Syst. 84, 371–386. 10.1007/s10846-015-0269-0

[B5] Deudon M. Cournut P. Lacoste A. Adulyasak Y. Rousseau L.-M. (2018). “Learning heuristics for the TSP by policy gradient,” in International Conference on the Integration of Constraint Programming, Artificial Intelligence, and Operations Research (Delft: Springer), 170–181. 10.1007/978-3-319-93031-2_12

[B6] Edison E. Shima T. (2011). Integrated task assignment and path optimization for cooperating uninhabited aerial vehicles using genetic algorithms. Comput. Operat. Res. 38, 340–356. 10.1016/j.cor.2010.06.001

[B7] Ferreira P. R. Jr. Boffo F. S. Bazzan A. L. (2007). “A swarm based approximated algorithm to the extended generalized assignment problem (e-GAP),” in Proceedings of the 6th International Joint Conference on Autonomous Agents and Multiagent Systems (Honolulu, HI), 1–3. 10.1145/1329125.1329373

[B8] François-Lavet V. Taralla D. Ernst D. Fonteneau R. (2016). “Deep reinforcement learning solutions for energy microgrids management,” in European Workshop on Reinforcement Learning (EWRL 2016) (Barcelona).

[B9] Holler J. Vuorio R. Qin Z. Tang X. Jiao Y. Jin T.. (2019). “Deep reinforcement learning for multi-driver vehicle dispatching and repositioning problem,” in 2019 IEEE International Conference on Data Mining (ICDM) (Seoul: IEEE), 1090–1095. 10.1109/ICDM.2019.00129

[B10] Hooshangi N. Alesheikh A. A. (2017). Agent-based task allocation under uncertainties in disaster environments: an approach to interval uncertainty. Int. J. Disaster Risk Reduct. 24, 160–171. 10.1016/j.ijdrr.2017.06.010

[B11] Huang H. Zhu D. Ding F. (2014). Dynamic task assignment and path planning for multi-AUV system in variable ocean current environment. J. Intell. Robot. Syst. 74, 999–1012. 10.1007/s10846-013-9870-222949070

[B12] Khan A. T. Li S. (2022). Smart surgical control under RCM constraint using bio-inspired network. Neurocomputing 470, 121–129. 10.1016/j.neucom.2021.10.116

[B13] Khan A. T. Li S. Cao X. (2021). Control framework for cooperative robots in smart home using bio-inspired neural network. Measurement 167, 108253. 10.1016/j.measurement.2020.108253

[B14] Khan A. T. Li S. Li Z. (2022). Obstacle avoidance and model-free tracking control for home automation using bio-inspired approach. Adv. Cont. Appl. Eng. Indust. Syst. 4, 1–14. 10.1002/adc2.63

[B15] Kool W. Van Hoof H. Welling M. (2018). Attention, learn to solve routing problems! arXiv preprint arXiv:1803.08475. 10.48550/arXiv.1803.08475

[B16] Li J. Zhang K. Xia G. (2017). “Multi-AUV cooperative task allocation based on improved contract network,” in 2017 IEEE International Conference on Mechatronics and Automation (ICMA) (Takamatsu: IEEE), 608–613. 10.1109/ICMA.2017.8015886

[B17] Ma Q. Ge S. He D. Thaker D. Drori I. (2019). Combinatorial optimization by graph pointer networks and hierarchical reinforcement learning. arXiv preprint arXiv:1911.04936. 10.48550/arXiv.1911.04936

[B18] MahmoudZadeh S. Powers D. M. Sammut K. Atyabi A. Yazdani A. (2018). A hierarchal planning framework for AUV mission management in a spatiotemporal varying ocean. Comput. Electric. Eng. 67, 741–760. 10.1016/j.compeleceng.2017.12.035

[B19] Page A. J. Keane T. M. Naughton T. J. (2010). Multi-heuristic dynamic task allocation using genetic algorithms in a heterogeneous distributed system. J. Parallel Distribut. Comput. 70, 758–766. 10.1016/j.jpdc.2010.03.011 20862190 PMC2927021

[B20] Ru J. Yu S. Wu H. Li Y. Wu C. Jia Z.. (2021). A multi-AUV path planning system based on the omni-directional sensing ability. J. Mar. Sci. Eng. 9, 806–827. 10.3390/jmse9080806

[B21] Solozabal R. Ceberio J. Sanchoyerto A. Zabala L. Blanco B. Liberal F. (2019). Virtual network function placement optimization with deep reinforcement learning. IEEE J. Select. Areas Commun. 38, 292–303. 10.1109/JSAC.2019.2959183

[B22] Vinyals O. Fortunato M. Jaitly N. (2015). “Pointer networks,” in Proceedings of NIPS 2015 (Montreal, QC), 2692–2700.

[B23] Xie B. Chen J. Shen L. (2018). “Cooperation algorithms in multi-agent systems for dynamic task allocation: a brief overview,” in 2018 37th Chinese Control Conference (CCC) (Wuhan: IEEE), 6776–6781. 10.23919/ChiCC.2018.8483939

[B24] Zhu D. Cao X. Sun B. Luo C. (2017). Biologically inspired self-organizing map applied to task assignment and path planning of an AUV system. IEEE Trans. Cogn. Dev. Syst. 10, 304–313. 10.1109/TCDS.2017.2727678

[B25] Zhu D. Zhou B. Yang S. X. (2020). A novel algorithm of multi-AUVs task assignment and path planning based on biologically inspired neural network map. IEEE Trans. Intell. Vehicles 6, 333–342. 10.1109/TIV.2020.3029369

[B26] Zhu D.-Q. Qu Y. Yang S. X. (2019). Multi-AUV som task allocation algorithm considering initial orientation and ocean current environment. Front. Inform. Technol. Electron. Eng. 20, 330–341. 10.1631/FITEE.1800562

